# Supplementary material for: Microbiome Structures and Beneficial Bacteria in Soybean Roots Under Field Conditions of Prolonged High Temperatures and Drought Stress
Source: Microorganisms. 2024 Dec 19;12(12):2630. doi: 10.3390/microorganisms12122630 (PMC11678281; doi:10.3390/microorganisms12122630)
Supplement: Supplementary file 1 [file microorganisms-12-02630-s001.zip › microorganisms-3359138-supplementary.pdf]

Supplementary Materials:

1. **Soybean field for bacterial isolation**

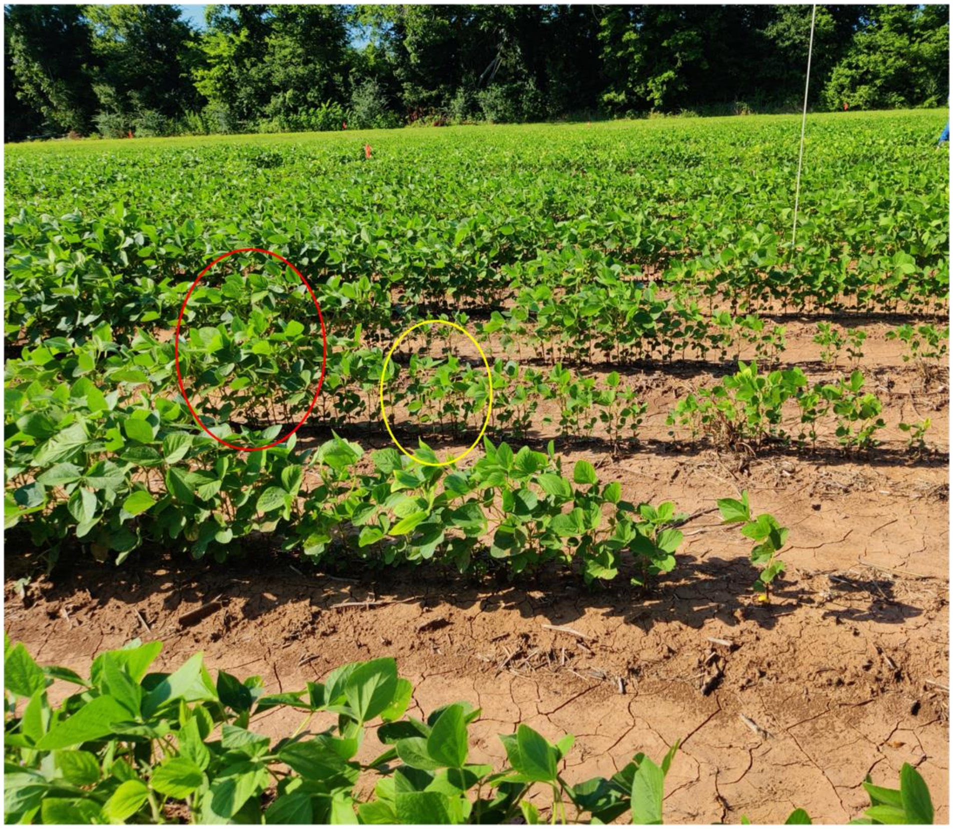

Figure S1: Selection of healthy soybean plants among a population of healthy (inside red circle) and stressed (inside yellow circle) plants under drought stress conditions

2. **Drought Stress Index (DSI) scoring guide:**

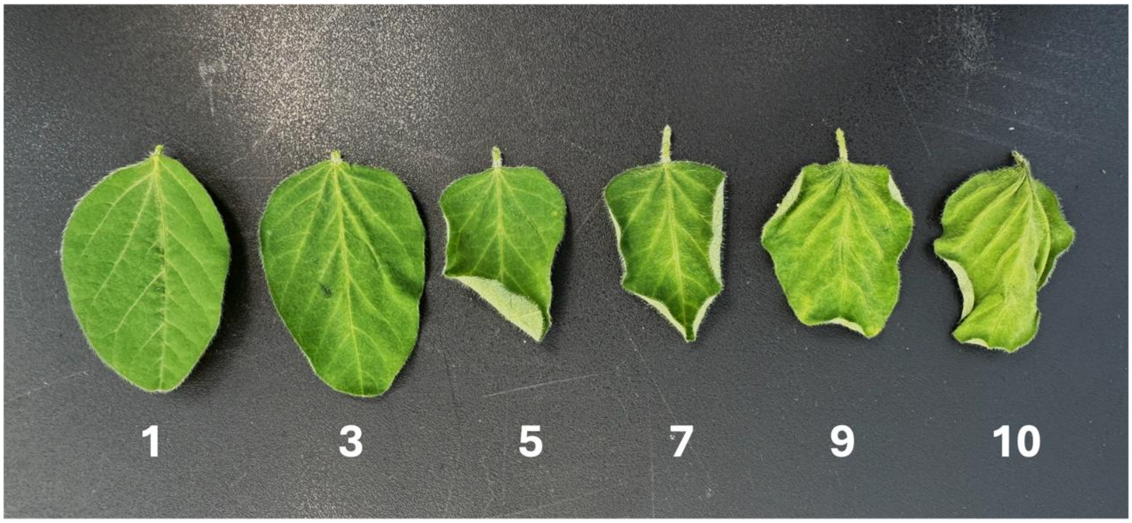

Figure S2: Picture showing the different status of leaves that correspond to the DSI scoring. a. Full fresh leaf (score 1), b. Slight loss of vigor with softness felt, no leaf rolling (score 3), c. Leaf partially rolled from one side only (score 5), d. Full leaf rolled inward from edges (score 7), e. Dry leaf starting to get crispy (score 9) and f. Fully dried leaf or crispy leaf (score 10)

3. Growth-promoting characteristics:

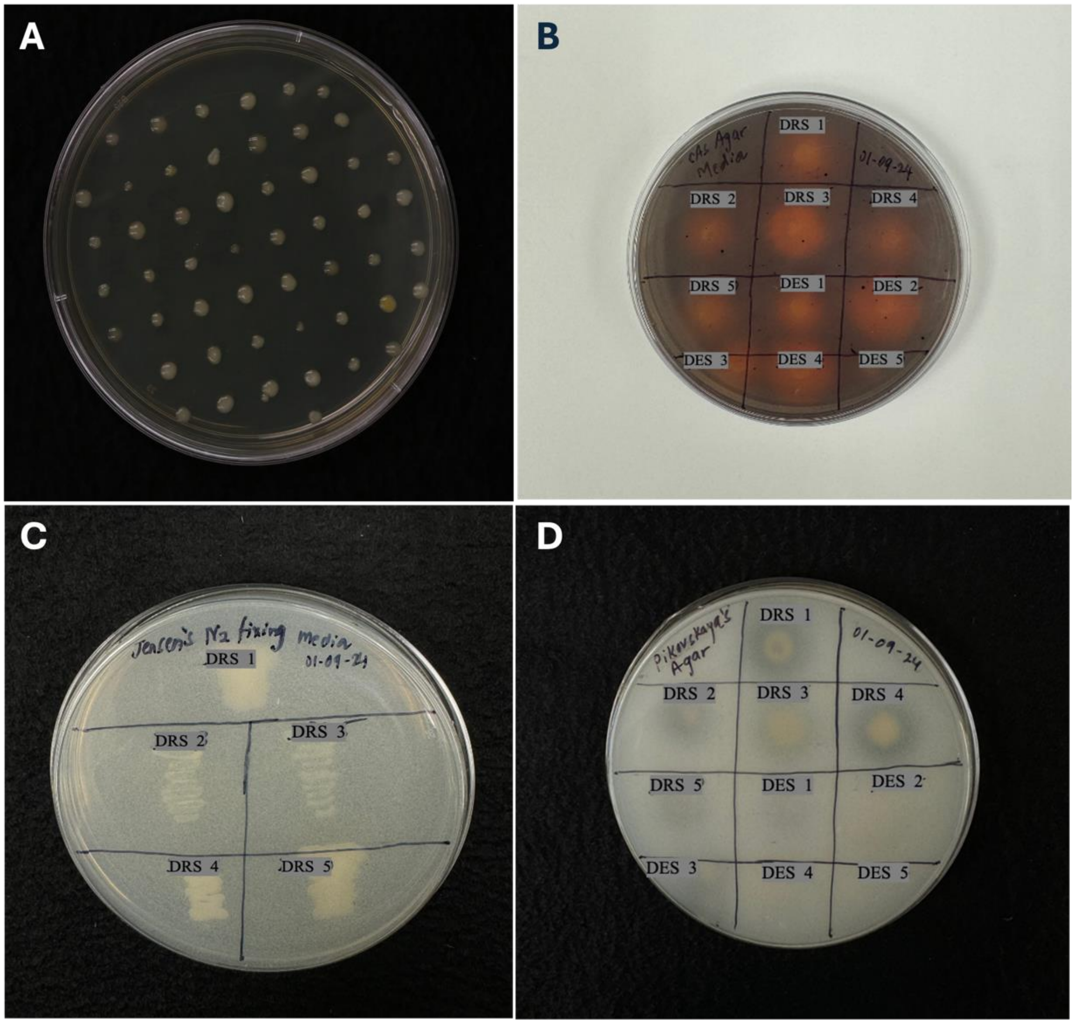

Figure S3: Assessment of growth-promoting characteristics of selected bacterial isolates. A. Single colony of bacterial isolates exhibiting mucoidal appearance on LB agar medium. B. Evaluation of siderophore production by bacteria on CAS agar medium. C. Assessment of atmospheric nitrogen-fixing capability of bacteria on Jensen's N2-free agar medium. D. Evaluation of phosphate solubilization by bacteria on Pikovskaya's agar medium.

4. Soybean seedling development under 7 days of drought stress

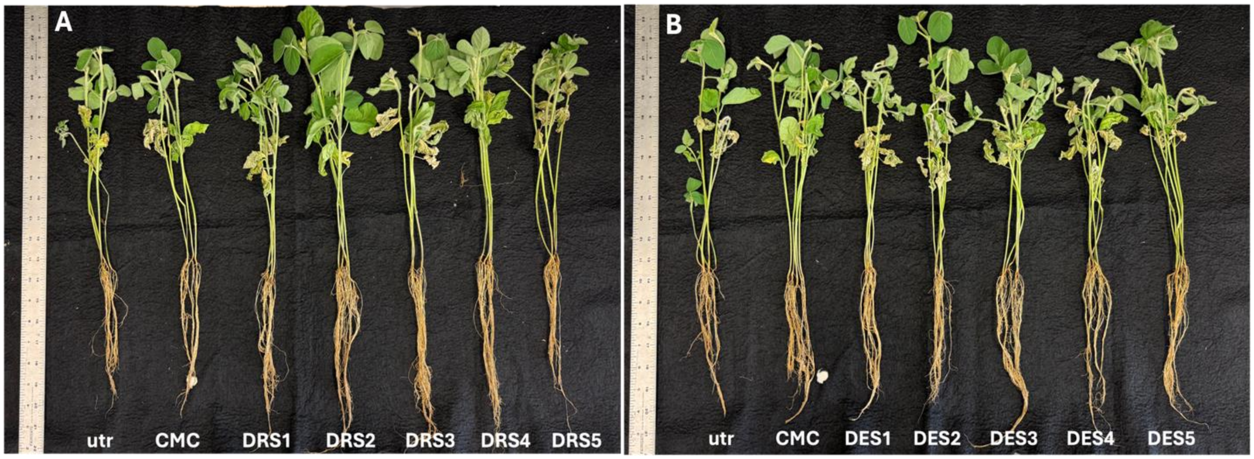

Figure S4: Representative images of soybean plants illustrating seedling development after 7 days of drought stress with various bacterial seed treatments
